# Constructing Sheet-On-Sheet Structured Graphitic Carbon Nitride/Reduced Graphene Oxide/Layered MnO_2_ Ternary Nanocomposite with Outstanding Catalytic Properties on Thermal Decomposition of Ammonium Perchlorate

**DOI:** 10.3390/nano7120450

**Published:** 2017-12-15

**Authors:** Jianhua Xu, Dongnan Li, Yu Chen, Linghua Tan, Bo Kou, Fushun Wan, Wei Jiang, Fengsheng Li

**Affiliations:** 1 National Special Superfine Power Engineering Research Center, Nanjing University of Science and Technology, Nanjing 210094, China; xujianhua931118@163.com (J.X.); joshldn@163.com (D.L.); climentjw@126.com (W.J.); lfs_njust@126.com (F.L.); 2 School of Chemical Engineering, Nanjing University of Science and Technology, Nanjing 210094, China; wanfushun0808@163.com; 3 Jiangsu Key Laboratory of Advanced Structural Materials and Application Technology, School of Materials Engineering, Nanjing Institute of Technology, Nanjing 211167, China; cy_5432@163.com (Y.C.); koutianbo@hotmail.com (B.K.)

**Keywords:** g-C_3_N_4_/rGO/MnO_2_, sheet-on-sheet, covalent coupling, in situ formation, synergistic effect

## Abstract

We unprecedentedly report that layered MnO_2_ nanosheets were in situ formed onto the surface of covalently bonded graphitic carbon nitride/reduced graphene oxide nanocomposite (g-C_3_N_4_/rGO), forming sheet-on-sheet structured two dimension (2D) graphitic carbon nitride/reduced graphene oxide/layered MnO_2_ ternary nanocomposite (g-C_3_N_4_/rGO/MnO_2_) with outstanding catalytic properties on thermal decomposition of ammonium perchlorate (AP). The covalently bonded g-C_3_N_4_/rGO was firstly prepared by the calcination of graphene oxide-guanidine hydrochloride precursor (GO-GndCl), following by its dispersion into the KMnO_4_ aqueous solution to construct the g-C_3_N_4_/rGO/MnO_2_ ternary nanocomposite. FT-IR, XRD, Raman as well as the XPS results clearly demonstrated the chemical interaction between g-C_3_N_4_, rGO and MnO_2_. TEM and element mapping indicated that layered g-C_3_N_4_/rGO was covered with thin MnO_2_ nanosheets. Furthermore, the obtained g-C_3_N_4_/rGO/MnO_2_ nanocomposite exhibited promising catalytic capacity on thermal decomposition of AP. Upon addition of 2 wt % g-C_3_N_4_/rGO/MnO_2_ ternary nanocomposite as catalyst, the thermal decomposition temperature of AP was largely decreased up by 142.5 °C, which was higher than that of pure g-C_3_N_4_, g-C_3_N_4_/rGO and MnO_2_, respectively, demonstrating the synergistic catalysis of the as-prepared nanocomposite.

## 1. Introduction

Recently, two-dimensional (2D) nanosheets have drawn increasing attention due to their special optical and electronic properties, as 2D nanosheets often show outstanding performance in catalysis [1], sensing [2], nanoelectronic devices [3] and so on. Among all the 2D materials, graphene is particularly attractive owning to its excellent thermal and chemical stability, large surface area and good electronic conductivity [4,5]. As an important derivative of graphene, graphene oxide (GO), which possesses many oxygen-containing functional groups on its basal planes, regarded as potential reactive sites for constructing 2D functional nanocomposite [6]. The prepared layered composite often has excellent properties owning to the interaction between the individual components. For example, by combining the respective advantages of SnS_2_ nanosheet (large interlayer spacing benefiting Na^+^ intercalation and diffusion) and reduced graphene oxide (rGO) (highly conductive network benefiting conduction of electron), the SnS_2_-rGO layered nanocomposite exhibited excellent electrochemical performance when was used as the anode of sodium-ion batteries [7]. Besides, due to intimate contact between WS_2_ nanosheet and rGO support, layered WS_2_/rGO nanocomposite synthesized via the hydrothermal reaction showed promising catalytic capacity for hydrogen evolution reaction [8]. Thus, it is of great value for constructing GO-based 2D nanomaterials. 

Among various layered materials, graphitic carbon nitride (g-C_3_N_4_) has similar carbon network and sp^2^ conjugated π structure compared to GO [9], which is considered as the most compatible material to couple with GO. In recent years, constructing of g-C_3_N_4_/GO or g-C_3_N_4_/rGO has been intensely studied. However, few of them focused on the design of covalently linked binary g-C_3_N_4_/GO or g-C_3_N_4_/rGO nanocomposite [10,11]. If g-C_3_N_4_ is covalently coupled with graphene sheets, the prepared composite would possess a very stable structure with excellent catalytic ability via the joint interaction between the individual nanosheets [12]. For example, Fu and his coworkers used an in situ synthetic approach to create a cross-linked g-C_3_N_4_/rGO composites that can be utilized as the anode of lithium-ion batteries with high, stable and reversible capacity [13]. Ong et al. [14] demonstrated that the sandwich-like graphene-g-C_3_N_4_ hybrid linked by C–O–C bond showed high visible-light photoactivity towards CO_2_ reduction. 

Coupling g-C_3_N_4_/rGO with another 2D materials to construct functional sheet-on-sheet structured ternary nanocomposite attracts substantial research efforts, which was motivated by the desire to combine the properties of each individual nanosheets [15]. Few-layered MnO_2_ nanosheet is known as a promising 2D material due to its potential application in many fields, such as supercapacitors [16], selective detection [17], tumor cell imaging [18], fluorescence sensor [19] and photocatalyst [20]. Such wide application made the preparation of MnO_2_ nanosheet draw great attention. Thus, it is of great significance to construct 2D g-C_3_N_4_/rGO/MnO_2_ ternary nanocomposite. 

Herein, we present the construction and characterization of sheet-on-sheet structured graphitic carbon nitride/reduced graphene oxide/layered MnO_2_ (g-C_3_N_4_/rGO/MnO_2_) ternary nanocomposite. Such unique architectures, which were the different nanosheets stacking with each other, could endow the material with excellent catalytic capacity. Furthermore, the as-prepared g-C_3_N_4_/rGO/MnO_2_ nanocomposite showed excellent catalytic performance on thermal decomposition of ammonium perchlorate (AP), a key energetic oxidizer in composite solid propellants [21]. To be best of our knowledge, there are not any reports regarding the synthesis and application of sheet-on-sheet structured g-C_3_N_4_/rGO/MnO_2_ nanocomposite till now.

## 2. Experimental

### 2.1. Synthesis of g-C_3_N_4_, g-C_3_N_4_/rGO and g-C_3_N_4_/rGO/MnO_2_ Ternary Nanocomposite

Guanidine hydrochloride (GndCl) and potassium permanganate (KMnO_4_) was purchased from Sinopharm Chemical Reagent Co., Ltd. (Beijing, China). Graphene oxide (thickness: 1~3 nm) was purchased from Nanjing XFNANO Materials Tech Co., Ltd. (Nanjing, China). All the chemicals were used without further purification.

Firstly, bulk g-C_3_N_4_ was synthesized by directly heating guanidine hydrochloride (GndCl) at 550 °C for 2 h in the tubular furnace under N_2_ atmosphere at a heating rate of 3 °C·min^−1^ [9]. 

Secondly, g-C_3_N_4_/rGO binary composite was prepared by an in situ method that g-C_3_N_4_ was covalently coupled with rGO. Typically, 100 mg graphene oxide was dispersed in 100 mL distilled water under ultrasonic sound (300 W) for 1 h. Afterwards, 300 mg GndCl was added into the above GO suspension and the mixture was heated at 80 °C under stirring for 4 h. Then, the grey sponge-like precursor (labeled as GndCl/GO) was collected by freeze-drying. Finally, the as-prepared precursor was placed into a tube furnace and calcined at 550 °C (N_2_ atmosphere) for 5 h at a heating rate of 3 °C·min^−1^ to obtain the binary composite labeled as g-C_3_N_4_/rGO (1:X). the X was the mass radio of the GndCl and GO. 

Thirdly, the g-C_3_N_4_/rGO/MnO_2_ ternary nanocomposite was prepared by adding 20 mg KMnO_4_ into 100 mL g-C_3_N_4_/rGO (1:3) binary composite suspension (0.4 mg/mL) and stirred at 80 °C for 2 days. The final production was collected by filtrating, washing and freeze-drying.

For comparison, pure MnO_2_ nanosheets without g-C_3_N_4_ and rGO were also obtained using the same method under the same condition. In detail, excess KMnO_4_ was added to the rGO suspension and stirred at 80 °C for 2 days. The resulted sample was collected by washing with deionized water and freeze-drying.

### 2.2. Characterization

The X-ray diffraction (XRD) patterns at wide (10–80° in 2θ) angles were collected using a Bruker D8 Advanced X-ray diffractometer (Bruker, Billerica, MA, USA) with Cu Kα irradiation (λ = 0.15406 nm). Fourier transform infrared (FT-IR) spectra of samples were recorded on Bruker Tensor 27 spectrometer (Bruker, Billerica, MA, USA). Raman spectrometer was conducted using a Laser Confocal inVia Raman Microspectrometer (Renishaw, UK) equipped with a 514 nm Ar ion solid excitation laser. The small peaks centered at 1120 cm^−1^ in all the Raman spectrum curve were assigned to the interference of fluorescence. X-ray photoelectron spectra (XPS) was performed using a Thermo Fisher ESCALAB250Xi instrument (Thermo Fisher, Waltham, MA, USA) with Al Kα radiation as the exciting source. The results obtained in the XPS analysis were corrected by referencing the C1s line to 284.8 eV. Transmission electron microscopy (TEM) images were obtained using a JEM-2100 microscope (JEOL, Tokyo, Japan) with an accelerating voltage of 100 kV. Energy-dispersive X-ray maps from the Scanning Transmission Electron Microscope were recorded using an FEI-Tecnai G2 F30 S-TWIN TEM (FEI, Hillsboro, OR, USA) operated at 200 kV. BET surface areas were obtained from 77 K N_2_ adsorption-desorption isotherms using a MICROMERITICS ASAP2020 surface area and porosimetry analyzer (Micromeritics, Norcross, GA, USA).

### 2.3. Catalytic Activity Measurement

To research on the catalytic performance of the as-prepared materials on the thermal decomposition of AP, a sample of AP mixed with g-C_3_N_4_, g-C_3_N_4_/rGO, MnO_2_ and g-C_3_N_4_/rGO/MnO_2_ were prepared, respectively.

Preparation of sample for catalytic research: 0.02 g of g-C_3_N_4_, g-C_3_N_4_/rGO, MnO_2_ and g-C_3_N_4_/rGO/MnO_2_ were mixed with 0.98 g of AP in ethanol at room temperature, respectively, stirred for 1 h and then dried at 60 °C for 1 h. 

The resulting sample was determined by DSC (American TA Instrument SDT Q600, American TA Instrument, New Castle, DE, USA) and TG (Beijing Hengjiu Instrumnet HTG-1, Beijing Hengjiu Instrumnet, Beijing, China) at a heating rate of 10 °C·min^−1^ over the range of 100–500 °C under N_2_ atmosphere.

## 3. Result and Discussion

**Scheme 1:**
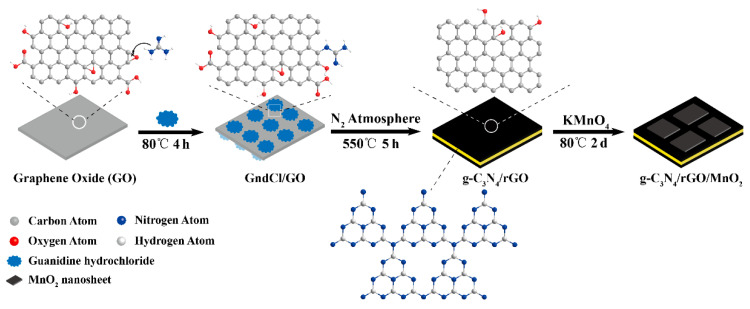
Illustration of the synthetic method of the g-C_3_N_4_/rGO/MnO_2_ ternary nanocomposite.

The overall synthetic procedure of the g-C_3_N_4_/rGO/MnO_2_ ternary nanocomposite was illustrated in Scheme 1. First, covalently coupled g-C_3_N_4_/rGO binary nanocomposite was fabricated through the in situ pyrolysis of guanidinium chloride (GndCl) on the surface of GO at 550 °C under nitrogen. During this process, water-soluble GndCl was firstly reacted with the active epoxy groups of GO via a nucleopilic substitution reaction. Upon calcining above precursor (GO-GndCl) under 550 °C, g-C_3_N_4_ was in situ prepared via the polymerization of GndCl. Meanwhile, nitrogen-containing species were released, which led to the partial reduction of GO. Subsequently, the as-prepared g-C_3_N_4_/rGO binary nanocomposite was dispersed in KMnO_4_ aqueous solution (80 °C) for two days. In this way, carbon atoms in the rGO framework would react with KMnO_4_ to in situ form MnO_2_ nanosheets via the partial depletion of rGO, which ultimately constructed g-C_3_N_4_/rGO/MnO_2_ ternary nanocomposite. 

**Figure 1:**
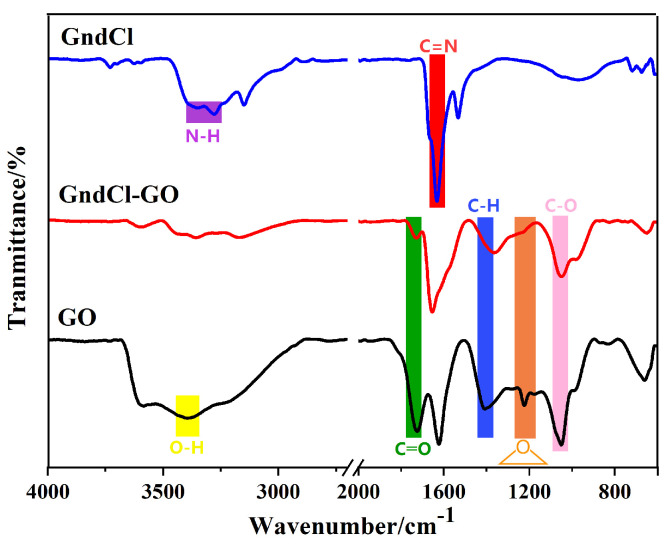
FT-IR spectra of GndCl, GO and GndCl-GO.

Figure 1 shows the FT-IR spectra of the GndCl, GndCl-GO and GO. Pure GO exhibited several absorption peaks in the range of 1000–2000 cm^−1^: C–OH (1051 cm^−1^), epoxide C–O–C (1221 cm^−1^), aromatic C=C (1628 cm^−1^) and C=O (1727 cm^−1^). It could be clearly found that the epoxide C–O–C peak of GndCl-GO at 1221 cm^−1^ was almost disappeared, which was attribute to the ring-opening reaction between NH_2_ (nucleophiles) and C–O–C group via an S_N_2 mechanism [13], demonstrating that GndCl was successfully grafted onto the surface of GO. 

**Figure 2:**
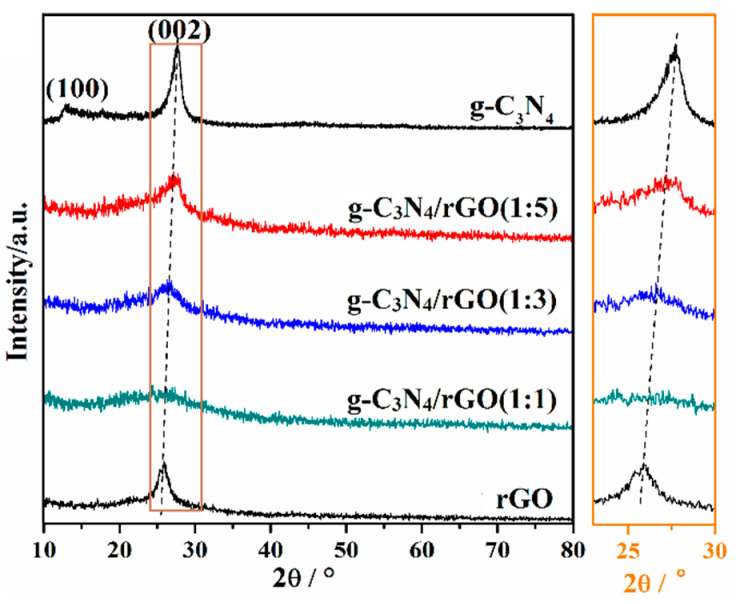
X-Ray Diffraction (XRD) diffraction pattern of g-C_3_N_4_, rGO, g-C_3_N_4_/rGO nanocomposite.

The as-prepared precursor (GO-GndCl) was then calcined to obtain the g-C_3_N_4_/rGO nanocomposite. Figure S1 showed the FT-IR curves of g-C_3_N_4_/rGO. It can be found that, apart from the absorption peaks belonging to g-C_3_N_4_, g-C_3_N_4_/rGO also exhibited an adsorption peak at 1558 cm^−1^, which could be assigned to the stretching vibrations of the graphene nanosheets [22]. This further confirmed the thermal reduction of GO under pyrolysis reaction. The crystal structure of the g-C_3_N_4_/rGO was investigated by X-ray diffraction (XRD). As shown in Figure 2, rGO exhibited a broad diffraction peak at 25.0°, corresponding to the inter-layer space about 0.36 nm of (002) plane. g-C_3_N_4_ contained two distinct diffraction peaks located at about 13.2° and 27.4°, which could be indexed of the (100) and (002) planes of hexagonal g-C_3_N_4_ (JCPDS 87-1526) [23], corresponding to the inter-layer stacking of aromatic segments with a distance of 0.33 nm and the in-plane structural packing motif, respectively. For g-C_3_N_4_/rGO nanocomposite with various content of g-C_3_N_4_, they only exhibited one diffraction peak between 25.0° and 27.4°, indicating a medium inter-layer distance compared to pure g-C_3_N_4_ and rGO, respectively, which could be ascribed to the stagger inter-layer stacking of g-C_3_N_4_ and rGO nanosheet.

**Figure 3:**
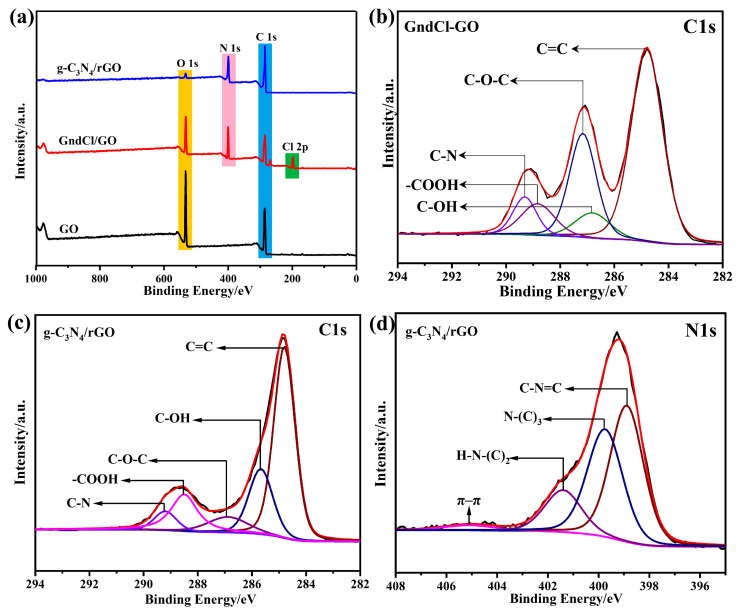
(**a**) XPS survey spectra of the GO, GndCl/GO and g-C_3_N_4_/rGO nanocomposite. (**b**) High resolution C 1s spectra of GndCl/GO precursor. High resolution C 1s (**c**) and N 1s (**d**) spectra of the g-C_3_N_4_/rGO nanocomposite.

**Figure 4:**
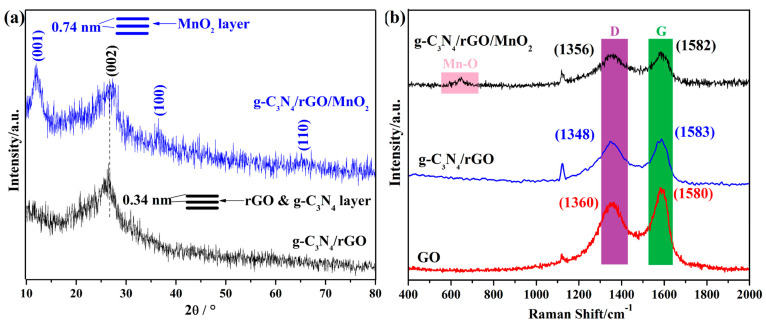
(**a**) XRD diffraction pattern of g-C_3_N_4_/rGO/MnO_2_ ternary nanocomposite. (**b**) Raman spectra of GO, g-C_3_N_4_/rGO and g-C_3_N_4_/rGO/MnO_2_ ternary nanocomposite.

X-ray photoelectron spectroscopy (XPS) was used to further study the element composition and chemical state of g-C_3_N_4_/rGO nanocomposite. From the data list in Table 1, the O content of g-C_3_N_4_/rGO was 9.13 wt %, which was lower that of pure GO (38.63 wt %), reflecting the partial reduction of GO via deoxygenation. Meanwhile, the presence of the introduction of g-C_3_N_4_ which have no oxygen also contributed to a decrease of O content in the g-C_3_N_4_/rGO composite. As shown in Figure 3a, compared with pure GO, the spectrum of GndCl-GO contained not only C and O element but also N element. Besides, as shown in Table 2 and Figure 3b, compared with pure GO, the epoxy group content of GO-GndCl was much lower, whereas hydroxyl groups content was remarkably increased. All these further support that GndCl was grafted onto GO via ring opening reaction, producing a large number of hydroxyl groups. The C1s spectra of the g-C_3_N_4_/rGO (Figure 3c) could be de-convoluted into five peaks located at 284.8 eV (C=C), 285.7 eV (C–OH), 286.9 eV (C–O–C), 288.6 eV (–COOH) and 289.3 eV (C–N). Except for the peaks belonging to rGO, it also contained the peak corresponding to C–N, suggesting the successful construction of g-C_3_N_4_/rGO nanocomposite. The N1s spectra (Figure 3d) provided further information regarding the formation of g-C_3_N_4_/rGO. It could be fitted into different peaks located at 398.9 eV, 399.8 eV and 401.4 eV, which could be assigned to sp^2^-hybridized pyridine nitrogen in triazine rings (C–N=C), tertiary nitrogen N–(C)_3_ groups and amino function ((C)_2_–N–H), respectively [24]. In addition, the peak at 405.1 eV was attributed to the charging effect of nitrogen-containing aromatic polymer [25], also reflecting the existence of g-C_3_N_4_ in the binary nanocomposite, which was in accordance with the C1s results discussed above.

g-C_3_N_4_/rGO/MnO_2_ ternary nanocomposite was synthesized via in situ formation of MnO_2_ nanosheet onto the surface of g-C_3_N_4_/rGO through oxidation-reduction reaction. Figure 4a showed the XRD spectra of g-C_3_N_4_/rGO/MnO_2_ nanocomposite, revealing the co-existence of g-C_3_N_4_, rGO and MnO_2_ in the ternary nanocomposite. The strong peak at 2θ = 12.0°, could be indexed as (001) of MnO_2_ nanosheet, indicating the layered hexagonal system, corresponding to a distance of 0.74 nm [26]. Meanwhile, the peak at 26.6° was assigned to inter-layer stagger stacking of the rGO and g-C_3_N_4_ nanosheet, which could be indexed as (002) plane with the distance of 0.34 nm [13]. In addition, the tiny peaks at 36.1° and 65.2° were indexed as (100) and (110) planes for the hexagonal 2D unit cell of MnO_2_ [16], which was also the evidence of the formation of layered MnO_2_ nanosheet. In order to further confirm the formation of g-C_3_N_4_/rGO/MnO_2_ nanocomposite, samples were characterized by Raman spectroscopy (Figure 4b). The appearance of small peak in the range of 550–700 cm^−1^, which could be assigned to Mn–O lattice vibrations [27], as well as the decrease of the D and G bands in the ternary nanocomposite demonstrate the yield of MnO_2_ nanosheet via partial depletion of rGO. The results of the XRD and Raman were in good agreement with FT-IR analysis (Figure S2). 

**Figure 5:**
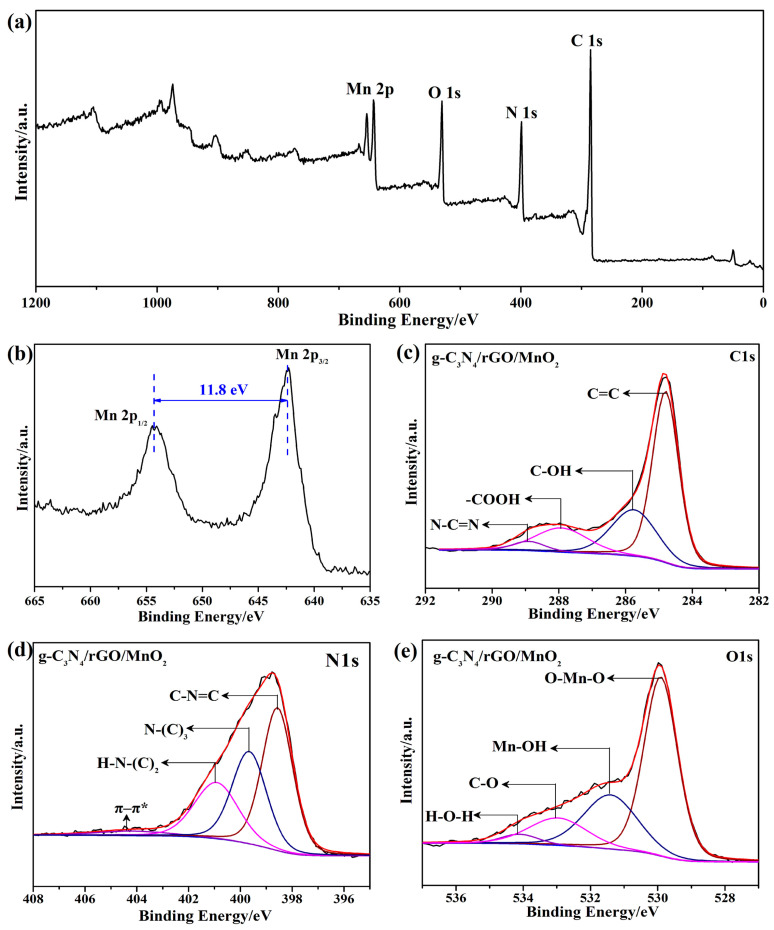
XPS spectra of the g-C_3_N_4_/rGO/MnO_2_ ternary nanocomposite: (**a**) full scan, (**b**) high resolution Mn 2p, (**c**) high resolution C 1s, (**d**) high resolution N 1s, (**e**) high resolution O 1s.

Figure 5a was the XPS survey spectra of g-C_3_N_4_/rGO/MnO_2_, which simultaneously contains C, N, O, Mn element, indicating the incorporation of Mn element into the framework of g-C_3_N_4_/rGO/MnO_2_. Furthermore, the separation of spin-energy between Mn 2p^1/2^ (654.4 eV) and 2p^3/2^ (642.4 eV) was 11.8 eV, confirming the existence of MnO_2_ [26] (Figure 5b). Figure 5c,d shows the high resolution C1s and N1s spectra of the g-C_3_N_4_/rGO/MnO_2_, respectively. Both the C 1s peak and N 1s peak could be fitted in four peaks, which were in accordance with the XPS analysis of g-C_3_N_4_/rGO nanocomposite, reflecting the existence of g-C_3_N_4_ and rGO in the ternary nanocomposite. However, being different from g-C_3_N_4_/rGO, the O1s spectra of g-C_3_N_4_/rGO/MnO_2_ could be de-convoluted into five peaks located at 529.9 eV, 531.4 eV, 533.0 eV and 534.2 eV (Figure 5e). The peaks at 529.9 eV and 531.4 eV were ascribed to the lattice of [MnO_6_] octahedra and the adsorbed oxygen in the presence of water molecular (Mn–OH) [16], respectively, which belong to MnO_2_ nanosheet. Besides, the peak at 533.0 eV was the typical C–O signal [12], which could be attributed to the presence of rGO.

**Figure 6:**
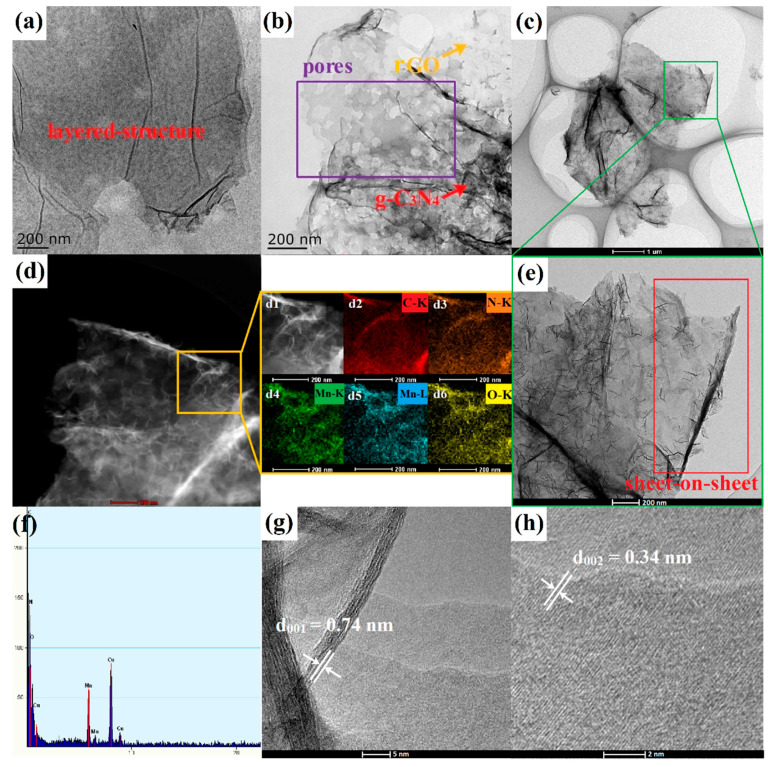
TEM images of (**a**) GO, (**b**) g-C_3_N_4_/rGO and (**c**,**e**) g-C_3_N_4_/rGO/MnO_2_ ternary nanocomposite. (**d**) elemental mappings, (**f**) EDS spectrum, (**g**,**h**) HRTEM images of the g-C_3_N_4_/rGO/MnO_2_ ternary nanocomposite. (inset images, corresponding height).

Transmission electron microscopy (TEM) study showed that pure GO exhibited layered structure with large aspect ratio (Figure 6a). Besides, as shown in Figure 6b, the as-prepared g-C_3_N_4_/rGO nanocomposite consisted of many pores, which could be attributed to gas removal (NH_3_, HCl) during pyrolysis process. Meanwhile, it could be clearly seen that g-C_3_N_4_/rGO showed a 2D layered structure with chiffon-like ripples and wrinkles. For g-C_3_N_4_/rGO/MnO_2_ nanocomposite (Figure 6c), the surface of g-C_3_N_4_/rGO was uniformly covered with MnO_2_ nanosheet with a sheet-on-sheet structure. Additionally, Figure 6e showed the enlarged TEM image of g-C_3_N_4_/rGO/MnO_2_. Notably, there was an intimate interfacial contact between MnO_2_, g-C_3_N_4_ and rGO nanosheet. To further illustrate the element distribution of the ternary nanocomposite, scanning transmission electron microscopy (STEM) experiment was carried out. Figure 6d was the STEM images of g-C_3_N_4_/rGO/MnO_2_ in dark field. Figure 6d1–d6 showed the element mapping of C, N, Mn and O, displaying the good distribution of MnO_2_ nanosheet on the interface of g-C_3_N_4_/rGO. Meanwhile, the energy dispersive X-Ray analysis (EDX) indicated the simultaneous presence of C, N, Mn and O (Figure 6f), which was in accordance with the analysis of element mapping. Figure 6g and h depicted the HRTEM images of g-C_3_N_4_/rGO/MnO_2_. As shown in Figure 6g, the lateral view showed the layered structure of the MnO_2_ nanosheet, giving a 0.74 nm lattice spacing of the (001) planes of MnO_2_. Besides, the HRTEM image in Figure 6h indicated a 0.34 nm basal spacing, corresponding to the (100) planes of g-C_3_N_4_/rGO nanocomposite. The TEM morphology characterization indicated that the g-C_3_N_4_/rGO/MnO_2_ were layer-structured nanosheets with large width-to-thickness ratio. 

**Figure 7:**
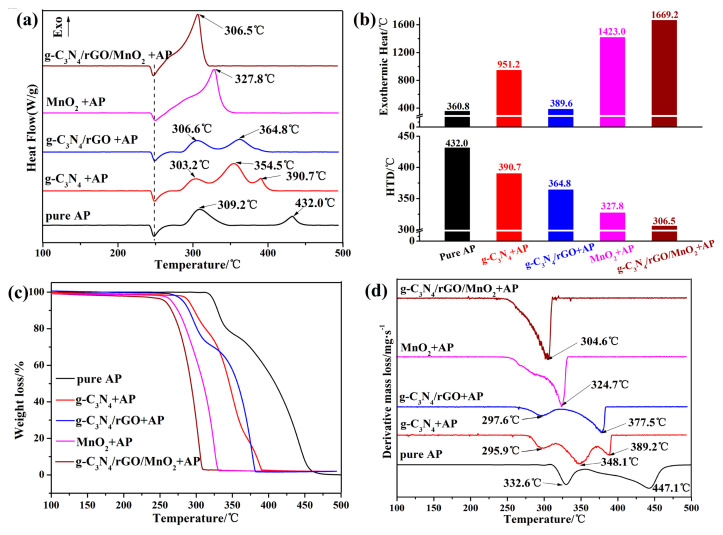
(**a**) DSC curves of AP in the absence and presence of catalysts. (**b**) histogram of the corresponding high decomposition temperature (HTD) and exothermic heat in Figure 7a. (**c**) TG and (**d**) DTG curves of AP in the absence and presence of catalysts. (the mass radio of catalyst to AP is 1:49).

The catalytic performance of g-C_3_N_4_/rGO/MnO_2_ ternary nanocomposite on thermal decomposition of ammonium perchlorate (AP) was investigated by DSC and TG and the results were illustrated in Figure 7. Figure 7a presented the DSC curves of AP in the absence and presence of 2 wt % g-C_3_N_4_, g-C_3_N_4_/rGO, MnO_2_ and g-C_3_N_4_/rGO/MnO_2_ catalyst, respectively. As shown, the thermal decomposition of pure AP contained two exothermic peaks: the high-temperature decomposition (HTD) centered at 309.5 °C and the low-temperature decomposition (LTD) centered at 432.3 °C. Besides, the endothermic peak at 248.3 °C was assigned to the crystal transformation process of AP [23]. The addition of different catalyst resulted in significant changes of the both high-temperature decomposition peak and low-temperature decomposition peak, whereas did not affect the crystal transformation temperature. Indeed, upon treated with 2 wt % pure g-C_3_N_4_ catalyst, the HTD peak of AP was decreased by 41.6 °C with the exothermic quantity of 951.2 J/g, which was similar to the previous reports [21], indicating the g-C_3_N_4_ was a promising catalyst for AP’s decomposition. Compared with the pure g-C_3_N_4_ catalyst, g-C_3_N_4_/rGO exhibited better catalytic performance than g-C_3_N_4_ alone and the HTD peak of AP could be decreased by 67.5 °C. This was attributed to the good conductivity of rGO, which could effectively accelerate the transfer of electron during the catalytic reaction [28]. For comparison, we also utilized rGO as templet to prepare pure MnO_2_ catalyst. As shown in DSC curves (Figure 7a), MnO_2_ also exhibited good catalytic ability on thermal decomposition of AP and the corresponding HTD peak was decreased by 105.2 °C with the exothermic quantity of 1423.0 J/g in the presence of 2 wt % MnO_2_. Notably, when AP was mixed with 2 wt % g-C_3_N_4_/rGO/MnO_2_ ternary nanocomposite, the HTD peak was remarkably decreased by 125.5 °C. Meanwhile, the exothermic heat of AP in the presence of this ternary catalyst was the highest comparing with other lines shown in Figure 7a, which was up to 1669.1 J/g (Figure 7b), demonstrating the promising synergistic catalysis. 

Figure 7c,d was the TG and DTG curves of the as-prepared samples, respectively. As displayed in Figure 7c, upon addition of g-C_3_N_4_ and g-C_3_N_4_/rGO binary nanocomposite as catalyst, respectively, the TG lines showed two weight loss steps from 100 °C to 500 °C, which was similar to the weight loss steps of pure AP in the absence of catalyst [29]. However, the two weight loss steps of AP were converted into a single step rapid decomposition process and the ending weight-loss decomposition temperature decreased in the presence of MnO_2_ and g-C_3_N_4_/rGO/MnO_2_ ternary nanocomposite, respectively, indicating that MnO_2_ and g-C_3_N_4_/rGO/MnO_2_ exhibited better catalytic performance than g-C_3_N_4_ and g-C_3_N_4_/rGO. Furthermore, in order to obviously distinguish the catalytic ability of MnO_2_ and g-C_3_N_4_/rGO/MnO_2_, we took the derivation of TG lines and the corresponding DTG curve was shown in Figure 7d. Specifically, the weight-loss decomposition temperature of AP in the presence 2 wt % MnO_2_ decreased by 122.4 °C, which was in accordance with the DSC analysis. However, when treated with 2 wt % g-C_3_N_4_/rGO/MnO_2_ ternary catalyst, the weight-loss decomposition temperature of AP was decreased by 142.5 °C, which was much higher than that of pure g-C_3_N_4_, g-C_3_N_4_/rGO and MnO_2_, separately. All these further suggested that the catalytic capacity of the ternary nanocomposite was remarkably enhanced via the synergistic effect of g-C_3_N_4_, rGO and MnO_2_. Based on DSC and TG analysis, we concluded that the as-prepared sheet-on-sheet structured 2D ternary g-C_3_N_4_/rGO/MnO_2_ nanocomposite exhibited excellent catalytic capacity on thermal decomposition of AP, which could be used as a promising catalyst for composite propellants in the future.

Why did the ternary g-C_3_N_4_/rGO/MnO_2_ exhibit excellent catalytic capacity than single g-C_3_N_4_ and MnO_2_ on thermal decomposition of AP? A previous study had demonstrated that the thermal decomposition process of AP consisted of two stages: low-temperature decomposition (LTD) process and high-temperature decomposition (HTD) process [30]. During the LTD process, a proton (H^+^) in AP transferred from the cation NH_4_^+^ to the anion ClO_4_^-^, forming NH_3_ and HClO_4_. After that, the generated HClO_4_ molecules decomposed rapidly and subsequently reacted with NH_3_. During HTD process, the generated NH_3_ and HClO_4_ NH_3_ in LTD process was completely oxidized by HClO_4_ in the gas phase.

Recently, He’s group demonstrated that g-C_3_N_4_ significantly affected the LTD process of AP [31]. In general, g-C_3_N_4_ possess a unique structure which was consisted of tri-s-triazine units connected by planar amino groups, which could be regarded as a Lewis base. During LTD process, the generated HClO_4_ would be firmly absorbed on g-C_3_N_4_ by the Lewis acid-base interaction, leading to the separation of HClO_4_ from the pores of AP. This was benefit to the proton transfer from NH_4_^+^ to ClO_4_^−^, accelerating the the decomposition process of AP [21]. 

Additionally, MnO_2_ was also a fantastic catalyst for AP’s thermal decomposition, confirmed by the DSC and TG data listed in Figure 7. As demonstrated by the previous literature, there were many lattice defects in the MnO_2_, in which existed large number of positive holes (h^+^) and electrons (e^−^) [29]. During the thermal decomposition process, the e^−^ was released and then reacted with HClO_4_ molecular to generate the superoxide radical anion ·O_2_^−^. Due to the power oxidation of the ·O_2_^−^ and h^+^, the NH_3_ would be further depleted to generate H_2_O, NO_2_ and N_2_O [32]. 

Reduced graphene oxide (rGO) was an excellent conductor, which could effectively accelerate the transfer of electron (e^−^), resulting in the faster decomposition of generated HClO_4_ molecular during the catalytic reaction [28]. Besides, the unique sheet-on-sheet structure was also beneficial for the acceleration of the transfer of e^−^ between different layers. In brief, by combination of the function of g-C_3_N_4_, rGO and MnO_2_, the ternary g-C_3_N_4_/rGO/MnO_2_ would promote the thermal decomposition process of AP.

## 4. Conclusions

In summary, a sheet-on-sheet structured 2D ternary nanocomposite (g-C_3_N_4_/rGO/MnO_2_) was successfully constructed via a two-step approach. In this procedure, FT-IR, XRD, Raman as well as the XPS were employed to investigate the formation process of the ternary nanocomposite. In general, GndCl was firstly grafted onto the interface of GO by ring-opening reaction to form GO-GndCl precursor and then the precursor was calcined, simultaneously leading to the formation of g-C_3_N_4_ and the reduction of GO, followed by the formation of covalently coupled g-C_3_N_4_/rGO nanocomposite. Secondly, layered MnO_2_ nanosheets were in situ formed onto the surface of g-C_3_N_4_/rGO via a facile redox method, constructing sheet-on-sheet structured g-C_3_N_4_/rGO/MnO_2_ ternary nanocomposite. The special layer-by-layer morphology ensured the excellent catalytic performance on thermal decomposition of AP. Upon addition of 2 wt % g-C_3_N_4_/rGO/MnO_2_ ternary catalyst, the thermal decomposition temperature of AP was decreased by 142.5 °C, which was higher than that of pure g-C_3_N_4_, g-C_3_N_4_/rGO and MnO_2_, respectively, demonstrating synergistic catalysis. Moreover, the fascinating structure of this g-C_3_N_4_/rGO/MnO_2_ ternary nanocomposite could widely expand its application in battery anode, supercapacitors, water oxidation and oxygen reduction, fluorescence sensor and so on.

## Figures and Tables

**Table 1:** C, O and N content (at%/wt %) of the sample according to XPS analysis from Figure 3.

Samples	C	N	O	Cl
GO	67.93/61.37	/	32.07/38.63	/
GndCl/GO	36.04/27.05	21.37/18.71	33.06/33.08	9.53/21.16
g-C_3_N_4_/GO	59.54/55.14	33.07/35.73	7.39/9.13	/

**Table 2:** C species content (at%) of the sample according to XPS analysis from Figure 3.

Samples	C=C	N–C=N	C–O–C	C–OH	–COOH
GO	48.01	/	44.57	2.97	4.45
GndCl/GO	51.95	7.56	23.97	7.51	9.02
g-C_3_N_4_/GO	51.61	5.31	7.89	19.80	15.40

## Supplementary Materials

The following are available online at https://www.mdpi.com/2079-4991/7/12/450/s1, Figure S1: FT-IR curves of the g-C_3_N_4_/rGO nanocomposite, Figure S2: FT-IR spectrum of the g-C_3_N_4_/rGO/MnO_2_ Ternary Nanocomposite.
